# SOX9: a key transcriptional regulator in organ fibrosis

**DOI:** 10.3389/fphar.2025.1507282

**Published:** 2025-02-05

**Authors:** Yishuo Li, Yue Xing, Ning Liu, Bin Liu, Zhihui Wang

**Affiliations:** Department of Cardiology, The Second Hospital of Jilin University, Changchun, China

**Keywords:** SOX9, fibrosis, cardiac fibrosis, liver fibrosis, renal fibrosis, pulmonary fibrosis

## Abstract

The *SOX9* gene locus is not only extensive but also intricate, and it could promote fibrosis in different organs or tissues, including cardiac fibrosis, liver fibrosis, kidney fibrosis, pulmonary fibrosis, as well as other organ fibrosis. Many disorders are associated with the process of fibrosis; moreover, fibrosis is a common symptom of chronic inflammatory diseases, characterized by the accumulation of excessive components in the extracellular matrix through different signaling pathways. The advanced stage of the fibrotic process leads to organ dysfunction and, ultimately, death. In this review, we first give an overview of the original structure and functions of SOX9. Second, we will discuss the role of SOX9 in fibrosis in various organs or tissues. Third, we describe and reveal the possibility of SOX9 as an antifibrotic treatment target. Finally, we will focus on the application of novel technologies for SOX9 and the subsequent investigation of fibrosis.

## 1 Introduction

**FIGURE 1:**
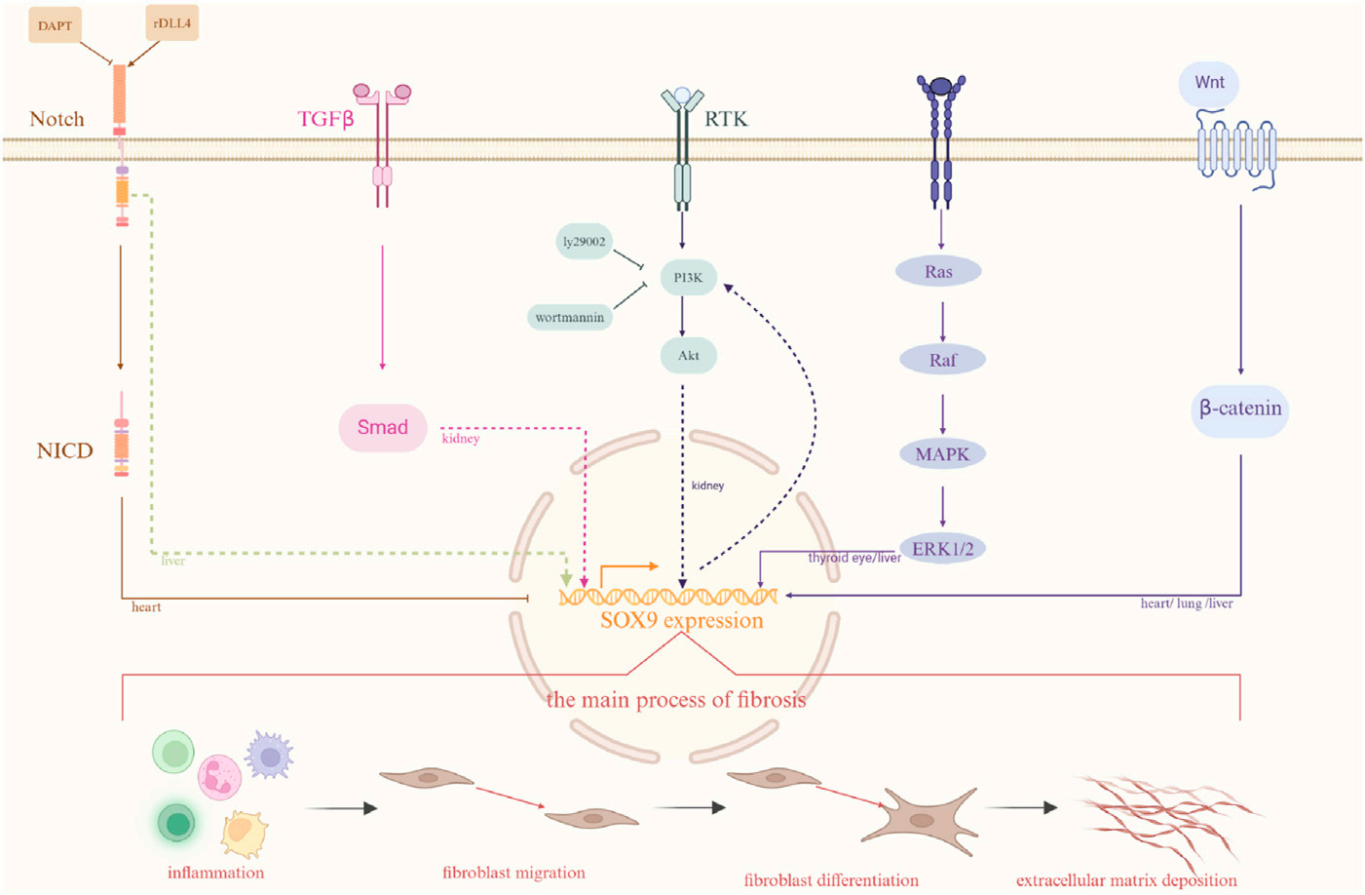
Different signaling pathways related to fibrosis. There are several different signaling pathways involved in fibrosis among different organ as well as the process of the development of fibrosis. The organs marked on each pathway in the diagram represent pathways that have been investigated in those organs. Solid lines indicate established interactions, whereas dashed lines imply potential molecular linkages. Created with BioRender.com.

SOX9 is a member of the SOX (SRY-associated high mobility group box) family of transcription factors. It impacts cell destiny by activating genes responsible for maintaining pluripotency, directing lineage differentiation, and stabilizing adult tissues. Concurrently, research in oncology is revealing SOX9’s involvement, and recent findings show its significant impact on fibrosis. Fibrosis can impact every organ and has traditionally been thought to be consistently progressive and irreversible. However, recent research in different organ systems has demonstrated that fibrosis is a dynamic process. The development of fibrotic tissue, marked by the excessive buildup of molecules like collagen and fibronectin, is actually a normal and crucial phase in organ tissue repair. Recurring or severe injuries often lead to a persistent buildup of extracellular matrix components, resulting in tissue disturbance, impaired organ function, and eventual organ failure. SOX9 may be the key regulators in fibrosis through different signaling pathways which has shown in Figure 1. This study offers a summary of the latest developments in comprehending the roles of SOX9 in fibrosis. Initially, we introduce the structure and function of SOX9. Additionally, we investigate the impact of SOX9 on the pathological mechanisms of fibrosis. Next, we discuss the prospective application of SOX9 target therapy in treating fibrosis.

## 2 SOX9

### 2.1 Location of SOX9

The *Sex-determining Region Y* (*SRY*) gene is situated on the Y chromosome and plays a role in sex determination (Berta et al., 1990). It has been identified as the crucial gene which is responsible for determining the sex of mammalian males and has a significant influence on the development of the testis. The *SRY* gene contains a DNA-binding region, the *High Mobility Group box* (*HMG-box*) domain, which is highly conserved, encodes 79 amino acids, and is analogous to the non-histone *HMG* in the chromosome (Bowles et al., 2000). Subsequently, there were many other additional transcription factors discovered that also had a domain that was very similar to the *HNG-box* of the *SRY* gene, leading to the collective name of the *SRY-related HMG-box* (*SOX*) gene family (Foster et al., 1994). Different biological and behavioral processes (Leung et al., 2020), such as the growth of the hair, heart, blood vessels, bone, and other tissues and organs, are all regulated by SOX. SOX plays a role in the regulation of these processes. There are twenty *SOX* genes that have been discovered in the genomes of both humans and mice, numbered A through H. A subgroup known as *SOXE* is comprised of *SOX9*, *SOX8*, and *SOX10* (Schepers et al., 2002; Wegner, 2010).

The *SOX9* gene has been mapped into mouse chromosome 11q and human chromosome 17q. In humans, the neighboring genes are positioned 2 megabases upstream and 0.5 megabases downstream, whereas in mice, the neighboring genes are located 1.7 megabases upstream and 0.5 megabases downstream.

### 2.2 Partners of *SOX9*

#### 2.2.1 Partners of promoter

The *SOX9* promoter resides upstream of the gene, in the DNA sequence preceding the transcription start site. Certain members of the protein family, cytokines, and fibroblast growth factors (TGF) may be involved in *SOX9* gene regulation mechanisms. Previous research has shown that fibroblast growth factors increase *Sox9* mRNA expression in mesenchymal C3H10T1/2 cell line and mice primary chondrocytes via MAP kinase-mediated pathway (Murakami et al., 2000). Furthermore, by chromatin immunoprecipitation and Dual-Luciferase reporter assay technology, through binding to the promoter of *SOX9*, the FOXO4 protein transcriptionally increases SOX9 expression, while IL-1β has the opposite effect (Ma et al., 2021). Recent research indicates that transcription factors CREB1 and CEBPB, which are attracted to the proximal promoter area, regulate the *SOX9* promoter in TM4 Sertoli cells (Diawara et al., 2023).

#### 2.2.2 Partners of enhancer

Numerous *SOX9* enhancers have been found. In mice, the *Testis-specific Enhancer of Sox9 (TES)* is a 3.2 kilobase (kb) enhancer, featuring a 1.4 kb core element (*TESCO*). This enhancer is situated 13 kb upstream of the *Sox9* gene, which could drive the expression of Sertoli cell-specific. *In vivo*, steroidogenic factor 1(Sf1) and SRY bind to *TES*, enhancing *Sox9* expression, and SOX9’s interaction with TESCO suggests a feed-forward regulation loop, maintaining its expression after SRY expression ceases (Sekido and Lovell-Badge, 2008). According to a different study, Sf1 binding to the *SOX9* gene enhancer is prevented by β-catenin activation during ovarian development, which suppresses *SOX9* expression and Sertoli cell differentiation (Bernard et al., 2012). *SOM*, an enhancer 70 kb upstream of mouse *Sox9*, works in tandem with *Sox9* dimers to activate *Sox9* expression, ensuring sufficient SOX9 molecules for *SOM* activation (Mead et al., 2013).

### 2.3 Modulations of SOX9

Modifications like methylation and acetylation of the *SOX9* gene promoter can regulate SOX9 expression levels. Many studies have demonstrated that DNA methylation can alter chromosome structure, DNA composition, stability, and DNA-protein interactions, thus controlling gene expression. During the fetal, neonatal, and adult periods of testicular development, no methylation was found in the *Sox9* gene (Pamnani et al., 2016). Furthermore, it was demonstrated that *CpG* sites 14 and 15 were totally methylated in the mature ovary, whereas site 16 was only 50% methylated (Pamnani et al., 2016). By methylating specific chromatin regions, the enzyme EZH2 contributes to the regulation of epigenetic gene expression (Li et al., 2023). Consequently, EZH2 binding to the *Sox9* promoter leads to chromatin compaction in this region, reducing *Sox9* expression and inhibiting its transcription (Li et al., 2023). DNA methylation is a common occurrence in differnet types of cancer. The *SOX9* gene’s promoter region is totally methylated in breast cancer, but it remains completely non-methylated in healthy cervical tissue (Wu et al., 2013). In gastric cancer, as the disease progresses, the methylation of the *SOX9* promoter significantly increases, potentially causing *SOX9* suppression in advanced stages (Sun et al., 2012). Additionally, in osteoarthritis, there is an observed increase in the trimethylation of H3K9 and H3K27 and H3K9, 15, 18, 23, and 27 had less acetylation at the *SOX9* promoters (Kim et al., 2013).

### 2.4 General properties of SOX9

**FIGURE 2:**
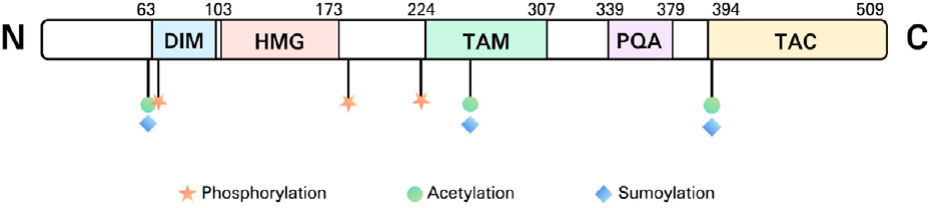
The structure of SOX9. DIM, dimerization domain; HMG, high mobility group; TAM, transactivation domains situated in the middle; PQA, proline/glutamine/alanine; TAC, transactivation domains situated in the C-terminus (TAC).

The human SOX9 protein, comprising 509 amino acids, features several distinct domains. These domains include a dimerization domain (DIM), an HMG box, a proline/glutamine/alanine (PQA)-rich domain, two transactivation domains situated in the middle (TAM) and at the C-terminus (TAC) of the protein (Figure 2).

SOX9, SOX8, and SOX10 are members of the SOXE subgroup, sharing homologous regions in the HMG, DIM, TAM, as well as TAC domains. The HMG domain is a DNA-binding region (with the SOX9 motif AGAACAATGG) that attaches to the minor groove of DNA, promoting sequence-specific DNA binding. This interaction bends the DNA into an L-shaped complex, altering the expression of the target gene (Mertin et al., 1999).

DIM is a structural domain present in SOXE transcription factors, located upstream of the HMG domain. The DIM domain is crucial for the formation of dimers in SOXE proteins. SOXE proteins can form both hetero- and homo-dimers via interactions between DIM and HMG, not via interactions between DIMs (Huang et al., 2015). In order to produce stable dimers, the DIM domain promotes contacts between SOXE proteins and helps them form selective dimers on non-compact DNA motifs (Huang et al., 2015).

The SOXE transcription factors contain two transactivation domains: TAM and TAC. Transactivation domains are protein segments that interact with other transcription factors, or co-activators, to boost gene transcription.

The PQA region is abundant in proline, glutamine, and alanine. It helps stabilize SOX9 and enhances transactivation *in vitro* while not having its own transactivation capabilities (McDowall et al., 1999; Haseeb and Lefebvre, 2019).

### 2.5 SOX9 protein post-translation modifications

Post-translational modifications (PTMs) of proteins are essential for diversifying protein functions and regulating signaling networks. SOX9 activity is regulated through various mechanisms, including PTMs.

#### 2.5.1 Serine phosphorylation

Three serine residues on SOX9 are phosphorylated: S64, S181, and S211. The phosphorylation of SOX9 at the S64 and S181 PKA sites occurs as a result of the activation of cAMP-dependent protein kinase A (PKA) during the development of gonadal development. This phosphorylation event enhances the binding of SOX9 to importin b, a protein involved in nucleocytoplasmic transport, resulting in nuclear localization (Malki et al., 2005). Extracellular signal-regulated kinases 1 and 2 (ERK1/2) are activated by sublytic C5b-9, which phosphorylates S64 and S181 in SOX9, plays an essential role in its proproliferative as well as profibrotic functions (Wu Z. et al., 2024). Another study showed that in chick neural tubes, extracellular signals induce the phosphorylation of S64 and S181 in SOX9, along with Snail2, leading to neural crest cell delamination (Liu et al., 2013). The phosphorylation site in S211 of SOX9 was identified in the research by Wendong Huang et al. Immunohistochemistry analysis of mouse embryo hind legs revealed the presence of phosphorylated SOX9 at S211 within the prehypertrophic region of the growth plate, a key site for parathyroid hormone-related peptide receptor expression (Huang et al., 2000).

#### 2.5.2 Lysine acetylation and deacetylation

The lysine residues at positions K61, K253, and K398 on SOX9 are subject to acetylation. Acetylation and deacetylation of SOX9 significantly affect the activation of genes that are specific to cartilage tissue. The presence of nuclear export signal (NES) and nuclear localization signal (NLS) promotes SOX9 localization to both the nucleus and the cytoplasm (Gasca et al., 2002; Südbeck and Scherer, 1997). According to a research, phosphorylation and acetylation of Sox9 are necessary for the sublytic C5b-9-induced proliferation of glomerular mesangial cells (GMCs) in rat Thy-1 nephritis (Thy-1N), which enhances Cyclin D1 gene transcription (Xie et al., 2021). The levels of SOX9 acetylation are closely linked to osteoarthritis development, as they diminish nuclear entry capability and impact transactivation of the *ACAN* gene (Bar et al., 2016). Deacetylase SIRT1 can remove acetyl groups from SOX9, facilitating its nuclear entry and activation. This suggests that regulating SOX9 acetylation levels and SIRT1 activity could be a potential treatment strategy for osteoarthritis (Bar et al., 2016).

#### 2.5.3 Ubiquitination

The ubiquitin proteasome system is the primary pathway for cells to degrade and eliminate unwanted proteins. Both *in vivo* and *in vitro*, E6-AP ubiquitinates SOX9 by attaching to it and acting as a ubiquitin ligase to bind ubiquitin to SOX9 (30). This ubiquitination leads to the degradation of SOX9, thus regulating its protein levels (Hattori et al., 2013). Additional research has been conducted on tumors. Cullin 3-based ubiquitin ligase KEAP1 regulates SOX9 protein stability via proteasome-mediated degradation (Shao et al., 2020). Loss-of-function mutations in KEAP1 impair its interaction with SOX9, inhibiting SOX9 ubiquitination, which results in elevated protein levels and promotes tumorigenesis (Shao et al., 2020). FBW7, an E3 ubiquitin ligase, is responsible for ubiquitinating and degrading specific target proteins. Rhabdomyosarcoma 2-associated transcript, a type of long-chain non-coding RNA, is shown in another tumor study to inhibit the development and dissemination of lung adenocarcinoma cells by mediating the ubiquitination and degradation of SOX9 (Pei et al., 2022). RMST directly binds to the SOX9 protein, enhancing its interaction with the ubiquitin ligase FBW7 (Pei et al., 2022).

#### 2.5.4 Lysine SUMOylation

SUMOylation, a post-translational protein modification, involves covalently attaching a small ubiquitin-like modifier (SUMO) to the substrate protein. SUMOylation can alter a protein’s structure, location, or stability. In human cells, the 61st, 253rd, and 398th lysines of SOX9 could be SUMOylated (Hattori et al., 2006). A study revealed that protein inhibitors of activated STAT (PIAS) contribute to the SUMOylation of SOX9, altering its function and stability. PIAS, functioning as a SUMO-binding enzyme, can conjugate SUMO proteins to SOX9, thereby increasing the stability of SOX9 by preventing its degradation (Hattori et al., 2006). Conversely, another study indicates that SUMOylation diminishes the synergistic activity of SOX9 with other co-factors, thus inhibiting its transcriptional activity (Oh et al., 2007). To measure SOX9-SUMOylation levels in live cells, researchers have developed a novel reporter gene, aiding in understanding SOX9’s regulatory dynamics during chondrogenesis (Saotome et al., 2020).

However, there is a paucity of studies concerning PTM in fibrosis. Fortunately, in other diseases, some authors have successfully achieved therapeutic effects by investigating the regulation of PTM of the SOX9 protein. In medulloblastoma, authors demonstrated that the phosphorylation of SOX9 could be degraded by FBW7. Ultimately, they demonstrate that pharmacological suppression of the PI3K/AKT/mTOR pathway destabilizes SOX9 in a GSK3/FBW7-dependent manner, enabling medulloblastoma cells susceptible to cytostatic therapy (Suryo Rahmanto et al., 2016). Jih-Yang Ko et al. have shown that in a model of transgenic mice that overexpressed human heat shock proteins 60 (TgHPS60), the overexpression level of HSP60 alleviated the pathological conditions of collagenase-induced osteoarthritis knees. Furthermore, the overexpression of HSP60 can sustain SOX9 levels and reduce the hyperubiquitination of SOX9 in the suffering joints (Ko et al., 2016). In another study about osteoarthritis, it shows that the PARylation (poly (ADP-ribosyl)ation), which promotes the ubiquitination and degradation of SOX9, is modified by tankyrase. They discovered that suppressing Tankyrase diminishes SOX9’s PTM (PARylation) and degradation, consequently augmenting its stability and boosting its transcriptional activity to stimulate the expression of cartilage matrix gene (Kim et al., 2019). Maybe in the future, research on fibrosis may yield advancements in disease therapy by concentrating on the PTM of SOX9.

### 2.6 SOX9 protein partners

SOX9 exhibits a variety of roles in different tissues by collaborating with protein partners to cooperatively activate and/or repress target genes.

Katsuhiko Amano et al. demonstrated that the AT-rich interactive domain containing protein 5a (Arid5a; also known as Mrf1), which was isolated from the ATDC5 cDNA library, physically engages with SOX9 in the nucleus and stimulates the chondrocyte-specific action of SOX9 (Amano et al., 2011). Peroxisome proliferator-activated receptor γ coactivator 1α (PGC-1α) and SOX9 are also closely related. PGC-1α acting as a coactivator of SOX9 contributes to chondrogenesis (Kawakami et al., 2005). In addition, Znf219 (zinc finger protein) colocalizes with SOX9 in the nucleus as well as having a physical association with SOX9 (Takigawa et al., 2010). They also found the overexpression of Znf219 also significantly enhanced the mRNA expression level of Col2α1, Agrecan, as well as Col11α2 (Takigawa et al., 2010). SOX9 primarily functions as a transcriptional activator, while it can also operate as a repressor. In another article, it has been shown that ZNF606, a novel co-regulator of SOX9, prevents chondrocyte differentiation through inhibiting SOX9 binding to the enhancers of gene col2α1 (Zhou et al., 2016).

## 3 SOX9 in organ and tissue development

**FIGURE 3:**
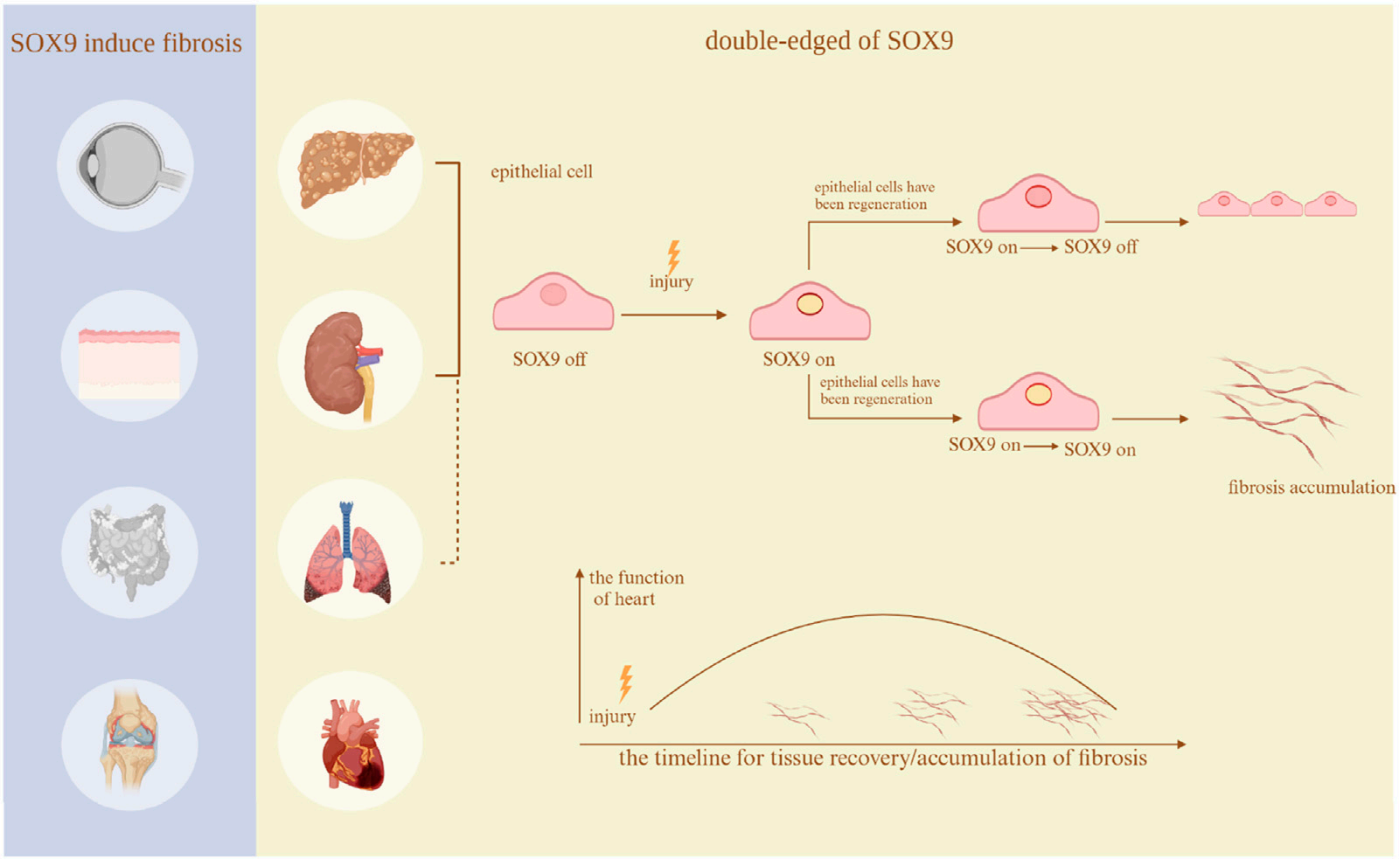
Fibrosis in different organs. The existing studies regarding the function of SOX9 in many organs. In thyroid eye disease, Crohn’s disease, arthritis, and skin disorders, SOX9 only exacerbates fibrosis. Nevertheless, in the heart, lungs, kidneys, and liver, it serves a dual function. The research regarding the lungs does not clearly show a dual role, hence it is depicted with a dashed line.Created with BioRender.com.

SOX9 has a variety of functions in the development of various organs, including sex determination (Gonen et al., 2018), chondrocyte differentiation (Liu et al., 2017), hair follicle formation (Vidal et al., 2005), pancreatic cell development (Seymour et al., 2007), prostate epithelial growth (Huang et al., 2012), and paneth cell formation in the small intestine (Bastide et al., 2007). Additionally, recent research has shown that SOX9 is an essential regulator of the fibrosis process (Figure 3).

### 3.1 Cardiac fibrosis

#### 3.1.1 The main procedure in cardiac fibrosis

The human heart consists of cardiomyocytes, non-cardiomyocytes (for instance fibroblasts, endothelial cells and vascular smooth muscle, and the extracellular matrix (Dostal et al., 2015). Due to the non-regenerative nature of cardiac myocytes, the replacement of dead cardiomyocytes following damage, such as a cardiac infarction, is crucial (Frangogiannis, 2012). Damaged areas trigger the inflammatory reaction, which clears matrix debris and dead cells and then activates the repaired procedures crucial for cardiac fibrosis (Frangogiannis, 2012). Cardiac fibrosis is characterized by the buildup of the extracellular matrix, particularly collagen I and III (Chikungunya Virus Infections, 2015). Various diseases (such as atrial fibrillation (AF), hypertension, heart failure, and myocardial infarction (MI)) can lead to cardiac fibrosis through different pathophysiological mechanisms, yet the process involves several cell types and similar cytokines (Kong et al., 2014).

In the fibrotic response, myofibroblasts (MFs) play a significant role. They can be differentiated from endothelial cells by the endothelium-mesenchymal transition (EndMT) and from cardiac fibroblasts (CFs), bone marrow-derived progenitors, and epicardial cells by the epithelial-mesenchymal transition (EMT) (Haudek et al., 2006; Zeisberg et al., 2007). Typically, CFs are inactive and do not synthesize proteins of the extracellular matrix (ECM). However, following cardiac injury, cytokines and growth factors mediate their transformation into MFs, which produce α-smooth muscle actin (α-SMA) (Hinz et al., 2007) indicating myofibroblast maturity (Shinde et al., 2017). MFs originating from bone marrow-derived precursors can produce collagen I, contributing to tensile strength (van Amerongen et al., 2008; Weber, 1989). EMT (Blom and Feng, 2018) and EndMT (Cheng W. et al., 2021) are regulated by numerous signaling pathways, including transforming growth factor β(TGF-β), wingless int (Wnt), Notch, and others, which are essential in heart fibrosis.

Immune cells are involved in the development of heart fibrosis. Tissue-resident macrophages are one of the initial immune cells that respond to indications of damage (Rurik et al., 2021). Due to their phagocytic properties, They can remove matrix debris, dead cells, and apoptotic MFs from the wound (Kong et al., 2014). Additionally, macrophages produce pro-inflammatory cytokines and chemokines, including CC-chemokine ligand 2 (CCL2), interleukin-6 (IL-6), and tumor necrosis factor (TNF) (Swirski and Nahrendorf, 2018). Mast cells can also secrete fibrogenic mediators, including histamine, cytokines, and growth factors (Levi-Schaffer and Rubinchik, 1994). Meanwhile, necrotizing cardiomyocytes release damage-related molecular motifs identified by neutrophils, dendritic cells, macrophages, and various other immune cells (Silvis et al., 2020). Those processes cause neutrophils and monocytes to migrate to the necrotic area, where they release proteolytic enzymes, MMPs, and factors such as IL-6 and TNF (Daseke et al., 2019). B lymphocytes produce inflammatory factors similar to those associated with fibrosis in neutrophils and monocytes (Cordero-Reyes et al., 2016). Additionally, neutrophils synthesize CCL, stimulating the expression of CCL and vascular cellular adhesion molecule (VCAM) in endothelial cells (Saparov et al., 2017). Endothelial cells are capable of generating pro-inflammatory chemokines and cytokines, which help attract lymphocytes and macrophages (Kong et al., 2014). These changes can further amplify inflammation (Saparov et al., 2017).

Cardiac fibrosis progresses due to a variety of cell types and signaling pathways. Numerous studies have identified the WNT and TGF-β signaling pathways as crucial in cardiac fibrosis. Researchers have found that these two signaling pathways can regulate the transformation of CFs (Yousefi et al., 2020).

#### 3.1.2 SOX9 in cardiac fibrosis

Recent research has shown a connection between cardiac fibrosis and SOX9. Cardiac fibrosis can be involved in various cardiovascular diseases, including hypertension, heart failure, AF, and MI. Studies suggest that SOX9 is involved in the pathogenic mechanisms associated with fibrosis.

As previously stated, EndMT is a major contributor to fibrosis. EndMT is crucial in the progression of cardiac fibrosis. This process involves transforming endothelial cells from their original state to mesenchymal-like phenotypes. Deleting *Sox9* in endothelial cells greatly inhibits EndMT as well as decreases scar formation during wound healing (Zhao et al., 2021). EJilai Zhao and colleagues, through mouse experiments, concluded that inhibition of Notch signaling upregulates *Sox9* gene expression in cells undergoing pathological EndMT, contributing to excess fibrosis (Zhao et al., 2021). Christopher L. Smith et al. reported that blood vessel formation and matrix organization require noncardiomyocytes (Smith et al., 2011). They identified SOX9 as the downstream transcription factor target in platelet-derived growth factor (PDGF)–stimulated EMT (70). Another study focused on endocardial fibroelastosis (EFE). Fibroblasts are mainly Fibroblasts primarily originate from epicardium-derived mesenchymal cells (MCs) (Zhang et al., 2017). Researchers discovered that in an EFE model, cells marked by SOX9 predominantly contribute to fibroblast formation (Zhang et al., 2017). In the EFE-like model, blocking TGF-β signaling could diminish tissue fibrosis and fibroblast accumulation (Zhang et al., 2017).

Following MI, cardiac fibrosis plays a role in myocardial remodeling. Recent studies have shown that reducing SOX9 expression effectively ameliorates fibrosis. Grégory P.A. Lacraz and colleagues employed tomo-seq and lineage-tracing experiments to identify genes co-regulated with cardiac fibrosis. Their findings revealed that most fibroblasts expressing COL1 originate from cells expressing SOX9 (Lacraz et al., 2017). And the fibrotic response of heart to ischemia damage was diminished when SOX9 was absent (Lacraz et al., 2017). In *Sox9* deficiency mice model, it confirmed that SOX9 is a common transcriptional regulator involved in ECM deposition and cardiac fibrosis mediation (Lacraz et al., 2017). Another team reached similar conclusions in a mouse model of MI, reporting that reducing SOX9 lessened the deposition of ECM and SOX9 also inhibited the activation of migrating fibroblasts *in vitro* (Scharf et al., 2019). Additionally, in fibroblasts, downregulating SOX9 expression can suppress severe inflammation in the infarct area, protect the heart from dilation, and improve cardiac function post-MI (Scharf et al., 2019). Adrián Ruiz-Villalba and colleagues found that SOX9 overexpression induces fibrosis-related gene expression in CFs, similar to the outcomes seen with TGF-β incubation (Ruiz-Villalba et al., 2020).

Myocardial fibrosis often occurs in the enlarged left ventricle in cases of arterial hypertension. Antje Schauer and colleagues showed that both control and *Sox9*-KO group developed high blood pressure, with the control group exhibiting fibrosis and left ventricular remodeling, while the conditional *Sox9*-KO group displayed delayed fibrosis and slight hypertrophy (Schauer et al., 2019). Interestingly, *Sox9* loss does not preserve cardiac function; instead, it slows down the development and remodeling of the heart.

Atrial fibrosis is crucial in the development of AF. A new research used cellular studies to demonstrate the function of SOX9 in human right atrial tissue. They isolated and cultured rat atrial fibroblasts, treating them with adenovirus to overexpress the *Sox9* gene or with siRNA to decrease SOX9 expression (Wang H. et al., 2021). Ultimately, they discovered that TGF-β1 increases SOX9 expression at the cellular level, and SOX9 overexpression may enhance cell migration, collagen deposition, and fibroblast differentiation (Wang H. et al., 2021). In patient atrial tissues, following RT-qPCR, Masson’s trichrome staining, and some other experimental methods, it was concluded that high expression level of SOX9 and α-SMA aggravates cardiac fibrosis (Wang H. et al., 2021).

Under conditions of cardiac pressure overload, SOX9 mediates hypertrophy and early fibrosis through cardiomyocytes and fibroblasts, ultimately leading to left ventricular remodeling. CHIA-FENG LIU and colleagues analyzed RNA levels in cardiac tissue samples from heart failure patients using whole-transcriptome sequencing, finding significant upregulation of *SOX9* RNA in patients with dilated cardiomyopathy and hypertrophic cardiomyopathy (Liu et al., 2022). They noted a significant association between *SOX9* RNA levels and the level of expression of fibrosis-related genes (Liu et al., 2022).

To sum up, overexpression of *SOX9* gene could induce cardiac fibrosis, several cell types are significant for tissue repair. However, excessive early inflammation can lead to severe degradation of the ECM, potentially causing heart rupture. Prolonged inflammation can diminish cardiac tensile strength and result in ventricular dilation. It is crucial to identify a balance that not only repairs damaged cardiac tissue but also preserves as much of the heart chamber’s function as possible.

### 3.2 Liver fibrosis

#### 3.2.1 The main procedure in liver fibrosis

The liver is composed of two main kinds of cells: parenchymal cells and non-parenchymal cells (NPLCs). Hepatocytes, a parenchymal cell type constituting the majority of liver cells, comprising about 60% of the overall cell count and around 80% of the volume of liver. Sinusoidal endothelial cells, Kupffer cells (a type of macrophage), and hepatic stellate cells (HSCs) are involved in NPLCs. Hepatic fibrosis, involving the aforementioned cells, can arise from various liver diseases. Hepatotoxic and cholestatic injuries represent the two prevalent forms of chronic liver damage leading to hepatic fibrosis. Hepatotoxic injury refers to long-term damage to liver cells, for instance, alcohol, metabolic syndrome including metabolic syndrome inducing hepatitis C virus (HCV) or hepatitis B virus (HBV) infections, non-alcoholic steatohepatitis (NASH). Cholestatic injury, caused by bile flow obstruction, can occur in conditions like primary sclerosing cholangitis (PSC), biliary atresia (BA), and primary or secondary biliary cholangitis (PBC).

Liver fibrosis results from repeated, persistent damage, starting with hepatocyte injury, leading to inflammation and the subsequent activation and recruitment of immune cells, resulting in the activation of HSCs. In response to injury or external stimuli, hepatocytes modify their gene expression and secretion profiles. When factors like TGF-β, Hedgehog, and Notch are expressed, they may activate HSCs. These cells then elevate α-smooth muscle actin levels, migrate to the damage site, and synthesize the ECM, forming a dense fibrous scar (Xu et al., 2016; Xie et al., 2013; Hu et al., 2022). In fibrotic livers, MFs which are absent in healthy livers, are the primary ECM source and become activated following liver injury (Kisseleva and Brenner, 2008). MFs, primarily responsible for collagen production and extracellular matrix accumulation, could originate from various sources. Inflammation also is essential to the pathogenesis of hepatic fibrosis, characterized by the recruitment of various inflammatory cells, including early activated proinflammatory macrophages, neutrophils, natural killer (NK) cells and monocytes. As initial responders, neutrophils are capable of chemotaxis and rapid migration to the site of inflammation, where they eliminate apoptotic hepatocytes. Experimental evidence indicates that neutrophil elimination exacerbates liver fibrosis, whereas their presence aids in fibrosis resolution (Calvente et al., 2019). The presence of various macrophage types is well-documented. Research has indicated that these cells may contribute to fibrosis by secreting substances including monocyte chemoattractant protein-1 and TGF-β (Kishore and Petrek, 2021). However, some studies have shown that macrophages may impede the fibrosis process. In the late stages of hepatic fibrosis, MMPs release can significantly influence fibrosis regression by affecting macrophages (Feng et al., 2018). NK cells, another type of immune cell, possess antifibrotic characteristics in liver fibrosis through the inhibition of HSC populations. NK cells have the ability to impact the fibrosis process through modulating the activity and cytokine secretion of other immune cells, such as macrophages and dendritic cells. NK cells directly kill activated HSCs by releasing cytotoxic molecules and inducing cell apoptosis, thereby reducing the progression of fibrosis (Tao et al., 2022). Maybe that was the difference between the liver fibrosis and the cardiac fibrosis. Liver regeneration typically occurs in cases of acute liver injury, such as liver resection and recovery from hepatitis (Campana et al., 2021). When the liver is acutely injured, hepatocytes undergo proliferation and differentiation to restore the damaged tissue. This regeneration process usually spans several days to weeks, allowing the liver to return to its normal size and restore functionality (Ben-Moshe et al., 2022). It is significant to note that the capacity of the liver to regenerate is restricted. In severe hepatic diseases, such as advanced cirrhosis, the regenerative ability of the liver may be impaired, leading to incomplete restoration. Another important phenomenon in liver fibrosis is the ductular response (DR), which is characterized by a proliferation of reactive bile ducts as a result of liver damage. Pathologically characteristic of biliary disorders such as BA, PSC, and PBC, DR is observed histologically in liver samples (Zhang Z. et al., 2022).

#### 3.2.2 SOX9 in liver fibrosis

In activated HSCs, *SOX9* was identified as an upregulated gene linked to liver fibrosis (Lin et al., 2020). There is a study concluded that SOX9 and β-catenin interactions may enhance HSC activation. The study revealed that SOX9 might bind to the TGF-β1 promoter, enhancing TGF-β1 transcription activity and thereby promoting HSC activation (Wang Q. et al., 2021). In the study by Varinder S. Athwal et al., it was found that over 30% of the genes (Osteopontin (OPN), et al.) regulated by SOX9 are involved in extracellular matrix production. Furthermore, RosaCreER: *Sox9* ^*fl/fl*^ mutant mice with the *Sox9* gene knocked down exhibited reduced fibrosis (Athwal et al., 2018). Another study shows that the PTM of histone H3 on the 18th lysine (H3K18) residue may regulate liver fibrosis. Later experiments demonstrated that the suppression of lactate dehydrogenase A (LDHA) obstructed H3K18 lactylation and positively regulated liver fibrosis by inhibiting HSC proliferation, motility, and ECM deposition (Wu S. et al., 2024). Furthermore, the overexpression of SOX9 significantly alleviated the effects of LDHA silencing on activated HSCs, suggesting that SOX9 operates downstream of H3K18 lactylation in the promotion of liver fibrosis (Wu S. et al., 2024).

Nonalcoholic fatty liver disease (NAFLD) is becoming increasingly prevalent. NAFLD advances from non-alcoholic fatty liver (NAFL) to NASH and might potentially result in liver cirrhosis and liver cancer. Z. Wang et al. used bioinformatics analysis on databases (GSE135251 and GSE162694) and concluded that *SOX9* genes are associated with liver fibrosis (Wang Z. et al., 2022). Another research group, analyzing databases (GSE89377 and GSE139602), found that elevated SOX9 expression is associated with heightened immune infiltration, suggesting its potential as a biomarker for cirrhosis diagnosis or therapeutic targeting (Du et al., 2023). Yue Li et al.'s research, utilizing the GEO database (GSE14323, GSE36411, and GSE89377), found a positive correlation between the degree of liver fibrosis and the levels of SOX9 expression (Li et al., 2022). Additionally, the higher expression level of SOX9 was found in hepatic fibrosis in mice (Li et al., 2022). Moreover, they demonstrated that patients with HCC had a considerably poorer 5-year survival rate than those without the disease by Kaplan-Meier curves (Li et al., 2022).

By using knockout technology, Zhu and colleagues were able to demonstrate how HSCs were stimulated by secreted phosphoprotein 1 (Spp1), which ultimately results in the generation of fibrogenic factors like Opn (Zhu et al., 2018). They noted that SOX9, a crucial transcriptional mediator in the signaling pathway, might enhance Spp1 expression during liver fibrosis progression (Zhu et al., 2018). Another fascinating study shows that c-Jun, an independent transcription factor, has cell-type-specific functions in mice. c-Jun helps protect hepatocytes from the regenerative DR of SOX9/Opn co-expressing NPLCs (Schulien et al., 2019). However, it regulates the expression of Opn and CD44 in NPLCs, which results in the promotion of fibrogenesis and DR (97). A high-fat diet (HFD) was shown to promote severe cholangitis in the research conducted by Shin Maeda and colleagues. This was attributed to the deletion of the E-cadherin gene (CDH1), which resulted in the absence of E-cadherin expression (Maeda et al., 2021). In these gene knockout models, the number of CD44 and SOX9-positive stem cell-like cells was vitally increased, which is associated with fibrosis (Maeda et al., 2021). The Cirrhosis Risk Score (CRS) gene includes SOX9, which acts as an indicator for liver progenitor cells. The Cirrhosis Risk Score (CRS) gene includes SOX9, which acts as an indicator for liver progenitor cells (Sanchez et al., 2023). SOX9 expression levels were significant predictors of hepatic fibrosis progression (Sanchez et al., 2023). In the choline-deficient diet supplemented with ethionine (CDE) injury model, SOX9-positive liver progenitor cells are significant in the fibrosis process (Li et al., 2024). Following CDE injury, there is a notable rise in the percentage of liver progenitor cells which exhibit SOX9 positivity inside the DR (100). Additionally, the expression of GITRL, a cytokine, is notably elevated in SOX9-positive hepatic progenitor cells, which are important in liver fibrosis (Li et al., 2024).

There are 9 genes (*ADAMTS2*, *ARHGEF5*, *CCT8*, *ERG*, *LBH*, *FRMD6*, *INMT*, *RASGRF2* and *DHRS4*) are related to SOX9 (Cai et al., 2025). All those 9 factors, except for *DHRS4*, which is protective, are considered deleterious and are recognized as risk factors in the progression of early-stage fibrosis generated by the HCV (Cai et al., 2025). Nourdine Hamdane et al. have been discribing that functional deletion of *SOX9*, which means selectively deleting or turning off a gene or gene segment in the genome, slows the growth of liver cancer cells. It was also found that SOX9 is overexpressed in liver tissues during chronic HCV infection, and this overexpression persists even after antiviral treatment (Hamdane et al., 2019). However, a different study revealed an alternate mechanism, concluding that HBV activates the promoter of *SOX9*, leading to the expression of SOX9 (103). Conversely, SOX9 inhibits the replication of HBV in human hepatoma cells by binding to and blocking HBV Enhll/Cp via HMG domain of *SOX9* gene (Yang H. et al., 2020). Fibrosis is associated not only with viral infections but also with aging. In E. G. Skurikhin et al.'s study, fibrosis induced by CCL4 or 5% ethanol may be associated with SOX9-positive cells (Skurikhin et al., 2021). In young rats, higher levels of SOX9-positive cells and MSCs activity lead to more severe liver fibrosis (Skurikhin et al., 2021).

Fibrosis is a reaction to liver damage, and cirrhosis represents the ultimate phase of fibrosis. Progression to cirrhosis result in increased mortality as well as an increased prevalence of hepatocellular carcinoma. Additionally, the SOX9/INHBB axis mediates the activation of HSCs, which produce collagen and the extracellular matrix, inducing liver fibrosis (Chen et al., 2021). Prolonged parasite infection, such as Clonorchis sinensis, can result in hepatic impairment, inflammation, and fibrotic responses. Without timely treatment, fibrosis can progress to more severe conditions, such as cirrhosis and liver cancer. According to immunohistochemistry and RT-PCR data, CK19, SOX9, and EpCAM, hepatic progenitor cell markers, were significantly upregulated in the experimental group (Qi et al., 2022).

Despite differing pathological mechanisms in the early stages of Alagille syndrome, BA, and cholestatic liver disease (CLD), these three diseases exhibit similar clinical symptoms. Numerous research has demonstrated the vital role of SOX9 in all three diseases. Manar M. Esmail and colleagues demonstrated that in a rat model of bile duct ligation, niclosamide may lessen liver fibrosis (Esmail et al., 2021). Furthermore, it has been shown that niclosamide lowers SOX9 expression in the NOTCH signaling pathway (Esmail et al., 2021). In CLD, the expression level of Hes1 could regulated by SOX9, and it could also associated with trans-differentiation of hepatocytes (Tharehalli et al., 2018). Duplication of the Rbpj gene can upregulate SOX9, which is associated with fibrosis (Tharehalli et al., 2018). In Dino Šisl et al.'s research, mice treated with treated with tamoxifen showed a much lower degree of liver fibrosis compared to those given a vehicle (corn oil) (Šisl et al., 2022). This conclusion was further supported by qPCR results showing low SOX9 expression, indicating inhibited activation of HSCs, biliary duct proliferation and collagen production (Šisl et al., 2022). Another study showed that reversine prevented liver fibrosis *in vitro* by causing HSC death, limiting cell proliferation, reducing HSC activation, and degrading the extracellular matrix (Huang et al., 2023). And they concluded that reversine inhibited cholestatic DR and liver fibrosis in rats, and decreased bile duct formation through the Dlk1/Notch/SOX9 signaling pathway (Huang et al., 2023).

BA leads to progressive scarring and damage of both extrahepatic and intrahepatic biliary ducts, resulting in obliterative cholangiopathy (Shamir et al., 2017). Giulia Jannone’s study showed that administering human allogeneic hepatogenic progenitor cells (HALPC) in biliary ligated mice successfully repaired biliary tract damage and reduced SOX9 expression levels (Jannone et al., 2023). In BA, numerous studies involving SOX9 have drawn conclusions from clinical patient biopsies. According to a study by Jenny E. Arboleda-Bustan et al. study, SOX9 was found to be highly expressed in the nuclei of reactive epithelium, fibrotic bridges and proliferating ductules in portal spaces (Arboleda-Bustan et al., 2022). In cholestatic liver injury, SOX9-positive liver progenitor-like cells (LPLCs) transform into cells with characteristics of bile duct cells, expressing markers like SOX9 (Lin et al., 2022). They also concluded that LPLCs can transform into reactive ductular cells (RDCs), which are associated with liver fibrosis, potentially leading to liver fibrosis when the liver is injured (Lin et al., 2022). However, another study demonstrated that SOX9-positive hepatocytes were identified in a bile duct ligation (BDL) mouse model. DR progression was quicker in wild-type BDL mice compared to liver epithelium-specific SOX9-knockout BDL mice (Yoshii et al., 2022). In other words, SOX9 facilitates the advancement of ductular responses to safeguard against chronic liver injury (Yoshii et al., 2022). An additional investigation, which examined liver specimens obtained from individuals diagnosed with BA, concluded that an important correlation among hepatic progenitor cells (HPCs) proliferation, aberrant SOX9 expression, and fibrosis in patients (El-Araby et al., 2021). Alagille syndrome is a genetic disorder causing severe liver disease. However, SOX9 levels are negatively correlated with severity, with lower levels in severe cases and higher levels in mild cases (Adams et al., 2020). A single *Sox9* copy conditionally deleted in *Jag1* ^*+/−*^ liver tissue reduces cholangiocyte biliary commitment and exacerbates liver fibrosis in Alagille syndrome (Adams et al., 2020).

Multiple mechanisms implicated in the regulation of hepatic fibrosis have been identified through the research mentioned above. These identified mechanisms have the potential to be therapeutic targets in the treatment of fibrosis. While there are no papers explicitly indicating that SOX9 is crucial for early healing in liver damage, continued expression in the later stages may lead to fibrosis. Nonetheless, we might hypothesize that the advantage of SOX9’s expression is dependent upon the timing of its expression.

### 3.3 Renal fibrosis

#### 3.3.1 The main procedure in renal fibrosis

The similar fibrotic process also occurs in the kidney. Renal fibrogenesis is often viewed as a failed wound healing process following initial damage from various injuries (Wynn, 2008). Crucial pathophysiological elements of both chronic kidney disease (CKD) and acute kidney injury (AKI) include renal inflammation and fibrosis.

The development of renal fibrosis requires the buildup of ECM components. MFs, originating from local fibroblasts exposed to fibrosis-promoting stimuli, are the primary contributors to ECM deposition in renal fibrosis. Other resident or infiltrating cells, including fibroblasts, tubular epithelial cells, mesangial cells, endothelial cells, podocytes, and macrophages, also contribute to matrix development. Fibroblasts are primarily residual in the kidney’s interstitium and are present in the normal cortex of a healthy kidney. In pathological conditions, pre-existing fibroblasts become activated and transdifferentiate into MFs. Similar to other organs, the kidneys also have multiple origins for MFs. Tubular epithelial cells and endothelial cells could differentiate into MFs through EndMT or EMT (Huang and Ogawa, 2020). However, there is considerable disagreement or debate surrounding this topic specifically in relation to the kidneys. The pericytes, also named perivascular fibroblasts, are described as the alternative origin for myofibroblast precursors (LeBleu et al., 2013). At the same time, both mesangial cells and podocytes can produce excessive pro-inflammatory and pro-fibrotic factors during renal fibrosis, stimulating the response of surrounding cells and matrix, and promoting the process of fibrosis (Song et al., 2023; Zhong et al., 2022).

The immune system is also involved in kidney fibrosis. Following an injury, chemotactic proteins create concentration gradients that attract inflammatory cells to the damaged site. Macrophages are usually the first immune cells to reach the site of inflammation. Macrophages have a dual function in renal fibrosis, as they contribute to both the progression of fibrosis and the inflammation reduction (Huen and Cantley, 2017). The chemokines as well as inflammatory mediators released by macrophages can attract other immune cells to the damaged tissues for participating in immune responses and repair processes.

#### 3.3.2 SOX9 in renal fibrosis

Renal fibrosis is a characteristic feature of CKD and commonly occurs in final stage of kidney disease, related to various types of renal damage and diminished kidney function. Numerous animal experiments have demonstrated a correlation between the expression of SOX9 and the development of fibrosis.

The high expression level of SOX9 has been found during renal fibrosis. Several potential genes including SOX9 have been found to be biomarkers for renal fibrosis by using datasets (GSE76882, GSE22459), and this was also confirmed in cell experiments by RT-qPCR (Guo et al., 2023). In both mouse models and biopsy tissues from CKD patients, increased expression level of SOX9 was also observed (Abe et al., 2015).

SOX9 is involved in multiple signaling pathways that regulate fibrosis. Sayyid Raza et al. have linked the transcription factor SOX9 to kidney fibrosis, suggesting it may regulate fibrosis via the SOX9-NAV3-YAP1 axis (Raza et al., 2021). Another signaling pathway indicates that SOX9 could enhance ECM synthesis and fibroblast activation. In Martina Feger et al.’s research, they found that during fibrosis, SOX9 expression is regulated by TGF-β1 as well as TGF-β-activated kinase 1 (Tak1) (Feger et al., 2015). TGF-β1 induces fibrotic effects by activating Smad-independent pathways (Feger et al., 2015). Tak1 is regarded as a key non-canonical signaling molecule in fibrosis which is regulated by TGF-β(127). Haixia Mao and colleagues found that serum levels of PIIINP, TGF-β1, and SOX9 were considerably elevated in a rat model of chronic kidney disease compared to the control group (Mao et al., 2023). SOX9 serum is anticipated to emerge as a novel diagnostic biomarker for renal fibrosis (Mao et al., 2023). Markus Tölle et al. demonstrated that uremic mice elevated levels of SOX9 expression in comparison to the control group (Tölle et al., 2022).

AKI is a life-threatening condition that can lead to CKD in survivors without specific treatment. In AKI, SOX9 expression has dual effects: promoting repair and exacerbating fibrosis. Different views have been demonstrated in the following articles. Kohei Matsushita and colleagues developed a rat model of AKI and investigated the immunohistochemistry markers of renal tubules throughout both adaptive and maladaptive restoration procedures (Matsushita et al., 2020). Following ischemia/reperfusion (I/R), both regenerating and dilated tubules could be observed in the AKI model, while regions of renal fibrosis demonstrate either tubular dilation or atrophy (Matsushita et al., 2020). Regenerative tubules progressively redifferentiated following I/R, while dilated tubules showed little capacity for redifferentiation (Matsushita et al., 2020). Immunohistochemical investigations revealed that regenerating tubules expressed survivin and SOX9, while fibrotic renal tubules also contained SOX9 (130). Therefore, SOX9 as well as survivin contribute to renal tubular regeneration, but persistent expression of SOX9 might be related to fibrosis (Matsushita et al., 2020). In other words, whether fibrosis occurs or whether severe fibrosis occurs is depend on the time-dependent expression of SOX9.

However, in the following studies, there is a disagreement on whether SOX9 will exacerbate fibrosis. Lu Yu et al. found that embryonic stem cell-derived extracellular vesicles (ESC-EVs), which could provide a therapeutic impact by stimulating SOX9^+^ cells in the kidney, notably enhanced the rebuilding of both the structure and function of the impaired kidney (Yu et al., 2021). They concluded that following kidney injury, *Sox9* activation can promote the proliferation and regeneration of renal tubular epithelial cells (Yu et al., 2021). Whereas, Hyun Mi Kang et al. concluded that SOX9 has the ability to control the expression of genes related to fibrosis, which affects the development and deposition of fibrous tissue (Yu et al., 2021). Shang Chen and their colleagues established an AKI model utilizing prostaglandin E2 (PGE2) medication in a SOX9 lineage tracing mouse model (Chen et al., 2024a). Their findings underscore the potential for activating endogenous renal SOX9^+^ stem cells by PGE2 for the regenerative treatment of AKI (Chen et al., 2024a). In models of AKI, the expression of microRNA (miR-1247) could downregulate the SOX9. In this article, authors suggest that the significant reduction in SOX9, an essential regeneration regulator, greatly exacerbates the severity of AKI and impairs cellular and molecular repair mechanisms following renal damage (Dreval et al., 2017). As AKI progresses, injury- and repair-regulated markers like SOX9, VCAM1, and EGR1 are upregulated in three new subtypes of proximal tubule cells (Zhang M. et al., 2022).

AKI damages kidney structures, leading to chronic inflammation, fibrosis, and damage to glomeruli and tubules, resulting in persistent renal tissue changes and ultimately CKD. A study showed that in a rat model of obstructive nephropathy, the expression levels of SOX9 were elevated in tubular epithelial cells (Zhang et al., 2020). EMT refers to the process whereby damaged renal tubular epithelial cells undergo a phenotypic transformation, adopting mesenchymal features and differentiating into fibroblasts. Additionally, it was shown that renal tubular EMT and ECM accumulation were enhanced by SOX9 overexpression in NRK-52E cells (Zhang et al., 2020). Furthermore, The research revealed that SOX9-mediated renal tubular EMT and ECM deposition may be regulated by the PI3K/AKT as well as TGFβ1 signaling pathway (Zhang et al., 2020). Xiao Wang et al. found that SOX9 is upregulated in the models of kidney injury. SOX9 exacerbates kidney injury development and progression by inhibiting miR-96-5p expression, which subsequently upregulates Trib3 expression (Wang et al., 2023). Additionally, SOX9 overexpression triggers the IL-6 signaling pathway, escalating the degree of kidney damage and the inflammatory response (Wang et al., 2023).

Within other various types of kidney diseases, a relationship between SOX9 and fibrosis persists. The study reveals that sublytic C5b-9 activates *Sox9* in GMCs, increasing the expression of fibroblast growth factor 1, PDGFα, and TGF-β1 (23). Overexpression of S64 and S181 in SOX9 significantly increases these genes, with phosphorylation modifications playing a crucial role (Wu Z. et al., 2024). In cultured mesangial cells, JQ1 can inhibit *Sox9* activation, regulating type IV collagen production associated with kidney fibrosis. Additionally, in the mouse model, JQ1 inhibits SOX9 nuclear translocation and interstitial fibrosis (Morgado-Pascual et al., 2022).

For many years, people have held differing opinions on whether SOX9 promotes fibrosis. Recently, Science magazine revealed the relationship between the SOX9 switch and fibrosis. They discovered a dynamic SOX9 switch in the healing of epithelial tissues. In models of AKI caused by ischemia-reperfusion injury and rhabdomyolysis resulting in CKD, there exist SOX9^on-off^ and SOX9^on-on^ lineages (Aggarwal et al., 2024). The SOX9^on-off^ lineage can inhibit SOX9 during regeneration, preventing fibrosis during healing, while the SOX9^on-on^ lineage sustains SOX9 activity throughout ongoing regeneration, which is intimately linked to myofibroblasts and results in fibrosis (Aggarwal et al., 2024). This study conducted in this article on the kidneys indicates that SOX9 is crucial for epithelial tissue repair; nevertheless, persistent expression may induce or worsen fibrosis.

In summary, the prolonged issue has now been resolved: SOX9 facilitates regeneration during the initial phases of kidney injury; however, persistent expression in later stages leads to fibrosis.

### 3.4 Pulmonary fibrosis and systemic sclerosis

#### 3.4.1 The main procedure in pulmonary fibrosis and systemic sclerosis

Pulmonary fibrosis, a clinical endpoint in various chronic lung disorders, including interstitial lung disease (ILD), idiopathic pulmonary fibrosis (IPF), and systemic sclerosis (SSc). It is characterized by persistent collagen deposition and recurrent lung damage (Noble et al., 2012; Kolb and Vašáková, 2019). Pulmonary fibrosis often develops following acute lung inflammation, triggered by factors like viral or bacterial infections, ionizing radiation, chemotherapy, and exposure to air irritants and pollutants (Kligerman, 2022). Alveolar macrophages, the first cells to encounter external pathogens and irritants, initiate and later resolve the immune response in the lungs. Proinflammatory cytokines and chemokines secreted by macrophages can increase chemotaxis and progressively enrich alveolar spaces with monocytes and neutrophils (Kligerman, 2022). MFs actively produce ECM components during lung tissue repair. In a healthy organism, MFs undergo apoptosis once a sufficient amount of ECM is formed. However, in the context of persistent inflammation, MFs evade programmed cell death, resulting in abnormal tissue repair, excessive ECM production, and ultimately pulmonary fibrosis. This process is similar to fibrosis in other organs.

Additionally, systemic sclerosis, an autoimmune disease, causes fibrosis and vascular abnormalities in multiple organs. The lungs are among the organs most commonly affected by systemic sclerosis. About 50%–65% of systemic sclerosis patients show interstitial lung abnormalities on HRCT. ILD is the primary cause of mortality among SSc patients (Volkmann et al., 2023). Owing to the shared characteristics of systemic sclerosis and pulmonary fibrosis, they are being addressed collectively.

#### 3.4.2 SOX9 in pulmonary fibrosis and systemic sclerosis

In tracheal injury, SOX9 expression was elevated in rat tracheal fibroblast cells under TGF-β1 treatment. SOX9 overexpression stimulated fibroblasts and enhanced ECM(143). Inhibition of SOX9 suppressed cell proliferation, migration, and ECM deposition and heightened apoptosis in rat tracheal fibroblast cells (Gu et al., 2024). Maybe there is a similar way related to kidney (the regeneration of SOX9 temporal switch triggers), but this article does not discover the specific mechanisms. Another group of people revealed the contrasting functions of interleukin-4 (IL-4) cytokine signaling in interstitial macrophages and type II alveolar epithelial cells (ATIIs) (Cai et al., 2024). IL-4Rα signaling in macrophages facilitates alveolar epithelial regeneration following bleomycin-induced lung damage (Cai et al., 2024). Through organ and murine models, they established that IL-4 directly influences the ATII subset, prompting the production of the transcription factor SOX9 and reprogramming it into a progenitor-like state with potential for alveolar lineages and airway (Cai et al., 2024). Kevin Y. Huang and colleagues showed that SOX9, PT63 et al. become activated during the differentiation of transitional from type 2 pneumocyte (AT2) cells to KRT5^-^/KRT17^+^ positive cells, closely associated with molecular phenotypes like fibrosis (Huang and Petretto, 2021). Prathibha R. Gajjala’s research shown that specifically deleting *Sox9* in fibroblasts during TGF-α-induced pulmonary fibrosis reduces collagen deposition and enhances lung function (Gajjala et al., 2021). Furthermore, in bleomycin-induced pulmonary fibrosis mouse model, they found that overexpressing SOX9 in MFs increases the activation of fibroblast as well as lead to lung fibrosis (Gajjala et al., 2021). In the silica-treated mouse model of silicosis, researchers found that SOX9 and SOX2 are markers of embryonic lung stem/progenitor cells (Zhou H. et al., 2024). During the early inflammatory phase of silicosis, SOX9 and SOX2 were re-expressed in the distal lung and exhibited abnormal distribution (Zhou H. et al., 2024). However, in the later stages of silicosis, SOX9’s expression level decreased and was unrelated to the progression of fibrosis, indicating that SOX9 could be important in regulating fibrosis during the development of silicosis (Zhou H. et al., 2024). In SSc, researchers have found that SOX9 is overexpressed in the lungs. Kristy M. Waldrep et al. demonstrated that insulin-like growth factor-II (IGF-II) induces SOX9 expression (Waldrep et al., 2023). In SSc lung tissues compared to healthy controls, there is an overexpression of SOX9 at both mRNA and protein levels (Waldrep et al., 2023). Through the IGF1R/IR hybrid receptor, IGF-II activates SOX9 in lung fibroblasts, and SOX9’s downstream effects include COL3A1, P4HA2, TGF-β2, and TGF-β3 (148). In Elisha D. O. Roberson et al.'s research, the *SOX9* locus is identified as a hub gene with significant connectivity in the systemic sclerosis skin gene network (Roberson et al., 2022).

Fibrosis can be a result of various other pulmonary disorders. The Wnt/β-catenin-SOX9 axis is activated in tracheal injury and fibrosis, and inhibiting SOX9 may ameliorate tracheal fibrosis (Gu et al., 2022). Milena Paw et al. suggested that overexpressing SOX9 activates human bronchial fibroblasts (HBFs) and enhances the transcription of genes associated with fibrosis *in vitro* (Paw et al., 2023). Additionally, SOX9 expression are increased by TGF-β1 stimulation and decreased by inhibition of TGF-β1 signaling pathway (Paw et al., 2023).

### 3.5 Fibrosis in other organs or tissues

Besides the previously mentioned organs, SOX9 has also been related to fibrosis in additional tissues or organs, such as the pancreas, gut, and joints. Due to limited research on SOX9 in these specific organs and tissues, only a concise overview is currently available.

Fibrosis, a key feature of frozen shoulder, is characterized by inflammation of the joint capsule and increased proliferation of fibrous tissue. According to Hiroaki Nishimoto’s research, patients with frozen shoulder had significantly higher levels of SOX9 expression (Nishimoto et al., 2022). In rotator cuff supraspinatus tendon injuries, researchers utilized *Sox9CreERT2* transgenic mice in lineage tracing experiments to investigate the fate of *Sox9lin* cells (Moser et al., 2018). They discovered that in the partial tear injury model, *Sox9lin* cells were mainly localized in distinct regions, especially in the enthesis fibrocartilage and tendon insertion cells (Moser et al., 2018). Chronic, extensive, and repetitive injuries can all contribute to fibrosis in rotator cuff injuries. The research by Alexander J. Vervaecke et al. demonstrated that arise in SOX9-positive fibrocartilage cells at the neo-enthesis site on day 7 after treatment with tendon progenitor cells (TPCs) (Vervaecke et al., 2022). However, this increase was not sustained, with no further increase in SOX9-positive cells observed on days 14 and 28 post-treatment (Vervaecke et al., 2022). This suggests that the effect of TPC treatment on SOX9-positive cells is temporary (Vervaecke et al., 2022). Another study concluded that scar-forming astrocytes (SAs) are a specific type of astrocyte that form scar tissue post-spinal cord injury (Tamaru et al., 2023). Researchers found that during the chronic phase of spinal cord injury, SAs express high levels of the *Sox9* gene. However, another study indicates that SOX9 expression can suppress fibrosis, but this may be attributed to varying environmental conditions in which SOX9 expresses. In this article, SOX9 may be part of a specific regulatory network, where it mainly acts on the normal differentiation of chondrocytes and the normal construction of cartilage matrix, rather than triggering fibrosis-related signaling pathways. Matrix mineralization as well as uncontrolled cartilage fibrosis typically impede the regeneration of cartilage. In the study of novel 3D hydrogel scaffolds, they have designed the scaffolds through multiple-step operations. The SOX9 expression plasmid is integrated into the specified region of the cellulose gel by the chitosan gene vector, thereby creating a biochemical regulatory zone and fulfilling its function (Pan et al., 2024). The scaffolds also contain Bone Marrow - derived Mesenchymal Stem Cells (BMSCs) in which chitosan was employed to (Pan et al., 2024). They concluded that the scaffolds they designed could prevent the fibrosis of chondrogenic cartilage and promote the regeneration of cartilage (Pan et al., 2024). Therefore, SOX9 may have a regulatory role in scar formation (Tamaru et al., 2023).

In Crohn’s disease (CD), TGF-β-induced *SOX9* mRNA expression was observed to decrease in human fibroblasts stimulated with a BCL2 antagonist, compared to the control group treated with a vehicle (Weder et al., 2018). The research revealed significant upregulation of SOX9 expression in vmp1 KO mice. Overexpression of SOX9 was associated with other pathological features of pancreatitis, including cell death, fibrosis, and infiltration of inflammatory cells (Wang S. et al., 2022).

In thyroid eye disease (TED), *SOX9* knockdown markedly reduces the contraction and antiapoptotic capacity of orbital fibroblasts (OFs), while SOX9 overexpression enhances the transformation, migratory, and proliferation abilities of OFs (Zhou M. et al., 2024). *SOX9* knockdown decreased the expression of phosphorylated ERK1/2, while its overexpression elicited the opposite effect (Zhou M. et al., 2024). The Knockdown of *SOX9* resulted in the downregulation of extracellular matrix-related genes, whereas SOX9 overexpression led to their upregulation (Zhou M. et al., 2024).

### 3.6 Research of SOX9 across various organs

At the same time, the overexpression of the extracellular matrix proteins may result in fibrosis in different organs. In the article of Yuto Yamauchi et al., have concluded that proline- and arginine-rich end leucine-rich repeat protein (PRELP) was shown to be elevated in hearts and livers affected by fibrosis and chronic inflammation (Yamauchi et al., 2024). In both cardiac and hepatic tissues, the knockdown of Prelp has been demonstrated to diminish collagen expression (Yamauchi et al., 2024). Felix A. Trogisch and their colleagues found that deletion of endothelial *Sox9* inhibited fibrosis and organ dysfunction in 2 mouse models of heart failure and also in models of lung and liver fibrosis (Trogisch et al., 2024). CCN2, a direct target of SOX9, is a crucial protein that may govern the activation of fibroblasts through endothelial cell (Trogisch et al., 2024). This suggests if SOX9 activity remains, pathological remodeling diminishes due to the cumulative release of profibrotic ligands.

## 4 Treatment of fibrosis

Fibrosis refers to an abnormal amount of ECM accumulated beyond the normal healing process to deal with tissue damage. In the heart, liver, and kidneys, research and drug development through SOX9 as a treatment target is already under way, but there are large gaps in this area in other organs (Table 1).

**TABLE 1:** Treatment in fibrosis of different organs.

Organ	Model	Intervention method	Treatment method	SOX9 expression	Fibrosis	References
Heart	Sunitinib induced CF in male Wistar albino rats	Sunitinib (300 mg/kg given over 4 weeks as 25 mg/kg orally, three times a week)	Sacubitril/valsartan (68 mg/kg/day orally) for 4 weeks	Reduce	Ameliorate	Mohamad et al. (2024)
Heart	ISO induce cardiac fibrosis mice	ISO (i.h. 5 mg/kg for 7 days)	Bellidifolin	Reduce	Ameliorate	Yao et al. (2022)
Heart	MI/RI models of rat		miR-30e	Reduce	Ameliorate	Cheng et al. (2021b)
Heart	LAD ligated induced MI rat		miR‐145	Reduce		Cui et al. (2021)
Heart	TCF21^MCM/+^;R26^EGFP^ mice		miR–129-5p	Reduce	Ameliorate	Medzikovic et al. (2023)
Heart	STZ-induced diabetes (cardiac fibrosis) mice	STZ (i.p.200 mg/kg)	Linagliptin	Reverse the low expression	Ameliorate	Adhikari et al. (2023)
Liver	CCL4/2-AAF induce HF rat	CCL4 (i.h. 50% CCl4-olive oil solution (2 mL/kg) twice a week for 8 weeks) 2-AAF (10 mg/kg/d)	YGJ (orally3.56 mg/kg)	Reduce	Ameliorate	Xu et al. (2021)
Liver	CCL4 induce HF mice	CCL4 (i.p. 50% CCl4 in olive oil solution twice a week for	CTL (gavage, 0.5, 1.5 and 4.5 g/kg 4 weeks)	Reduce	Ameliorate	Dong et al. (2024)
Liver	HFD induce diabetes mice	HFD (4 weeks)	Cordycepin (orally 10 mg/kg/day or 20 mg/kg/day 12 weeks)	Reduce	Ameliorate	Chen et al. (2024b)
Liver	DMN induce HF rat CCl4 treated Sox9CreERT2ROSA26: YFP mice	DMN (i.p. 10 mg/kg body weight) CCl4 (0.5 μL/g body weight)	siRNA SP47 (i.v. 5 times or 10 times in DMN group i.v. 10 times or 20 times in CCl4 group)	Reduce	Ameliorate and regeneration	Sato et al. (2021)
Liver	HFD and fructose induce NASH rat	HFD (60% fat +1% cholesterol +0.25% cholic acid) and 20% w/v fructose in drinking water	EMPA (orally, 30 mg/kg/day)	Reduce	Ameliorate	Elseweidy et al. (2023)
Liver	CCL4 induce HF mice	CCl4 (0.4 mL/kg, twice a	miR-15a	Reduce	Ameliorate	Fu et al. (2022)
Kidney	UUO or folic acid-induced mice renal fibrosis	Folic acid (i.p. 250 mg/kg dissolved in 300 mM NaHCO_3_)	miR-30	Reduce	Ameliorate	Li et al. (2018)
Kidney	MI/RI models of rat		hAD-MSCs and their released exosomes	Increase	Ameliorate	Zhu et al. (2017)

Abbreviation: i.h., subcutaneously; ISO, isoproterenol; STZ, streptozotocin; i.p., intraperitoneal MI/RI, myocardial ischemia reperfusion injury; miRNAs, microRNAs; LAD ligated, left anterior descending coronary artery; TCF21^MCM/+^;R26^EGFP^ mice, Tracking and labeling CF expressing the TCF21 gene; MI, Myocardial infarction; CF, cardiac fibroblasts; HFD, high-fat diet; 2-AAF, 2-acetylaminofluorene; CCl4, carbon tetrachloride; YGJ, Yiguanjian decoction; DMN, dimethyl-nitrosamine; siRNA HSP47, VA-liposome siHSP47; Sox9CreERT2ROSA26: YFP, knockout Sox9 gene; DMSO, dimethyl sulfoxide; NASH, Non-alcoholic steatohepatitis; HFD, high fat diet; EMPA, Empagliflozin; HF, hepatic fibrosis; miR, microRNA; hAD-MSCs, human adipose-derived mesenchymal stem cells; UUO, unilateral ureteral obstruction; CTL, Carthamus tinctorius L; HFD, high-fat diet; CF, cardiac fibrosis.

Multiple studies have been conducted to investigate many promising avenues for combating cardiac fibrosis. Sacubitril/valsartan reduces sunitinib-induced cardiac fibrosis and oxidative stress by enhancing the targeting thioredoxin-interacting protein/thioredoxin system and downregulating the NF-ĸB/Wnt/β-catenin/SOX9 signaling pathway (Mohamad et al., 2024). Bellidifolin, an active component that prevents cardiac damage, could ameliorate cardiac fibrosis by inhibiting SOX9, thereby impeding the expression of α-SMA, collagen III and collagen I (163). MicroRNAs are involved in cell differentiation, apoptosis, proliferation, as well as pathological processes like cardiac fibrosis (Oltra et al., 2020; Yang JX. et al., 2020) and hold potential as innovative treatments. Nan Cheng et al. conducted experiments on an animal model with myocardial ischemia-reperfusion injury (MI/RI) as well as a cell experiment treated with hypoxia-reoxygenation injury (H/R), concluding that elevated microRNA-30e could attenuate MI/RI and induce ventricular remodeling via SOX9 downregulation (Cheng N. et al., 2021). Another research group investigated the relationship between microRNA-145 (miR-145) and SOX9 in MI(167). Shengyu Cui et al. found that miR-145 was suppressed in the boundary region between infarction and distinct areas, but SOX9 was overexpressed there (Cui et al., 2021). They also found that miR-145 mediated MI through SOX9 downregulation via the AKT/GSK-3β/β-catenin signaling pathway (Cui et al., 2021). In the study by Lejla Medzikovic and others, it was confirmed that cells in human hearts with calcification and myocardial fibrosis had higher levels of ASPN and SOX9 as well as lower levels of miR-129-5p (Medzikovic et al., 2023). Similar to the impact of reducing the expression of SOX9 and ASPN, they observed that miR-129-5p inhibited the transition of CF-to-MF and CF-to-osteogenic fibroblasts (OF) in primary cardiac fibroblasts (Medzikovic et al., 2023). Furthermore, the cardiac fibrosis caused by diabetes could also be ameliorated by decreasing the process of EndMT. Notably, one study highlights that SOX9 serves a protective function. Under diabetes conditions, molecules associated with necroptosis are increased and activated, coinciding with the suppression of the *Sox9* gene (Adhikari et al., 2023). However, the administration of a dipeptidyl peptidase (DPP)-4 inhibitor called linagliptin has been shown to reverse the downregulation of SOX9 expression, which may have a potential role in reducing cardiac fibrosis (Adhikari et al., 2023).

In CCL4-induced hepatic fibrosis in mice, SOX9 expression, crucial for activating HSCs in liver fibrosis development, significantly increases. Depleting *Sox9* or *H19* using antisense oligoribonucleotides (ASO) has demonstrated efficacy in preventing liver fibrosis (Wang et al., 2020). Yiguanjian, a traditional Chinese medicine, has been shown to be an effective medicine for liver cirrhosis in individuals with a liver-kidney yin deficit (Xu et al., 2021). Another study found that Yiguanjian could reduce SOX9 expression in a liver fibrosis rat model and potentially prevent the differentiation of hepatic progenitor cells into MFs, thereby alleviating liver fibrosis (Xu et al., 2021). Carthamus tinctorius L. (CTL) is a multifunctional cash crop. A variety of chemicals, such as alkaloids and flavonoids, have been extracted and characterized from CTL(172). In CCL4-induced hepatic fibrosis, CTL could diminish the level of alanine aminotransferase levels as well as aspartate aminotransferase and enhanced E-cadherin expression while reducing the expression of collagen I, α-SMA, SOX9, and hydroxyproline (Dong et al., 2024). Another Chinese traditional herb, Cordycepin, could ameliorate hepatic fibrosis, which is caused by diabetes, via inhibiting the activation of the SOX9-mediated Wnt/β-catenin pathway (Chen et al., 2024b). A study has explored the reduction of liver fibrosis. After VA-liposome siHSP47 therapy, the fibrotic liver in *Sox9* mutant mice showed regeneration capabilities (Sato et al., 2021). Recent studies have identified a cell type in the adult liver known as “chemically induced liver progenitors.” By using certain culture conditions as well as tiny chemicals, it is possible to induce these cells from mature liver hepatocytes through self-renew or differentiated into diverse kinds of liver cells. In Takayuki Miyoshi et al.'s experiment, liver tissues from surgically resected livers were analyzed, leading to the conclusion that SOX9 is a marker of liver progenitor cells associated with hepatic fibrosis (Miyoshi et al., 2022). In a zebrafish model, liver progenitor cells (LPCs) were induced to differentiate into hepatocytes by blocking the EGFR-MEK-ERK-SOX9 signaling pathway and then replacing damaged hepatocytes. Without this inhibition, LPCs release proinflammatory cytokines, exacerbating fibrosis and inflammation (So et al., 2021). These findings, regarding liver progenitor cells, open up new possibilities for liver regeneration and the treatment of liver diseases. Empagliflozin (EMPA) is an oral medication used to treat diabetes. Additionally, studies have shown that EMPA can restore normal liver functions and reduce the levels of inflammatory cytokines in the liver. That results demonstrate the anti-fibrotic effects of EMPA on fibrosis of liver via downregulating the NF-κB/SOX9/OPN signaling pathway (Elseweidy et al., 2023). MicroRNA also has substantial implications for the therapy of hepatic fibrosis. A report indicated that miRNA, which could potentially treat fibrosis, showed that miR-15a targets *Sox9* possibly inhibiting the development of hepatic fibrosis (Fu et al., 2022).

Additionally, SOX9 expression is inhibited by miR-30, which is regulated by TGF-β, acting as a downstream inhibitor as well as therapeutic microRNA for kidney fibrosis (Li et al., 2018). Another study discovered that using human adipose-derived mesenchymal stem cells (hAD-MSCs) and their released exosomes can stimulate the expression of SOX9 in tubular epithelial cells (TECs) (Zhu et al., 2017). This activation prevents TECs from transforming into a fibrotic phenotype, thus reducing the progression of renal fibrosis. Research also revealed that inhibiting exosome release or SOX9 expression in TECs can reverse this anti-fibrotic effect (Zhu et al., 2017).

## 5 Summary and outlook

The role of SOX9 has the potential to be the target therapy in various fibrosis. It is important to note that SOX9 has a role in pathophysiology of organ fibrosis. SOX9 is well recognized as a double-edged sword and its expression during regeneration and progressive fibrosis requires delicate balance. Sustained SOX9 expression has been demonstrated to negatively impact tissue regression and facilitate progressive fibrosis. Nonetheless, SOX9 exhibits significant attributes as a marker for progenitor cells implicated in regeneration and healing. Recent research indicates that a spatial and temporal switch triggers regeneration, and the dysregulation of this switch may result in an overactive healing response, subsequently leading to fibrosis. Shikhar Aggarwal et al. have demonstrated that the relevance between SOX9 transcription factor with the process of tissue remodeling as well as the progression of disease. The article shows that the diverse characteristics of the SOX9 transcription factor, which affirm its potential as a target for fibrosis treatment. However, recent studies about SOX9 do not show delicate organ-specific as well as the interactions among other molecular pathways in fibrosis.

**FIGURE 4:**
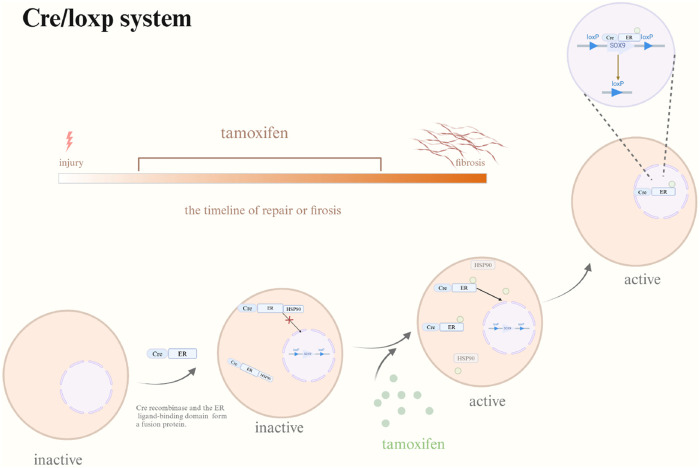
Cre/loxp system. Abbreviation: Cre, Cre recombinase; ER, ER ligand-binding domain; HSP90, heat shock protein 90; Tamoxifen may be provided at any point to investigate the impact of SOX9 gene knockout on tissue regeneration and fibrosis under varying temporal situations. Cre/loxp system triggered by Tamoxifen. In the absence of tamoxifen, Cre-ER demonstrates a binding affinity for HSP90 which is localized in the cytoplasm. Following tamoxifen application, it moves HSP90 and associates with Cre-ER, leading to the nuclear translocation of Cre-ER and the eventual inactivation of the target gene. Created with BioRender.com.

Fortunately, numerous gene editing methods are currently advancing. Considering that SOX9 expression is in a temporally manner, the recently developed gene editing method CRISPR/Cas9 may be inappropriate for the investigations of SOX9. Cre/loxp system (Figure 4) has more advantageous for the analysis of SOX9. Researchers can administer tamoxifen to different models of disease, throughout different periods to examine the impact of suppressing SOX9 expression on tissue healing and fibrosis. Furthermore, the Cre/loxp system has also overcame the issue of embryonic lethality or premature death following the knockout of essential genes, providing a reasonable and effective scientific means for further investigation. At the same time, with the application of pathway inhibitors and activators, regulating the expression of SOX9 to further observe tissue regeneration as well as fibrosis, which may help us in analyzing specific mechanisms of diseases progression.

SOX9 has the potential to serve as a biomarker for early detection of early regenerative responses. The development of SOX9 in the first phases of tissue injury may facilitate tissue repair (including the kidneys and liver), while diminishing SOX9 expression last stage can enhance patient prognosis. Future research might integrate SOX9 studies with prospect fields like bioinformatics and systems biology for a more thorough understanding of its role in fibrosis. Such an interdisciplinary method may uncover novel interactions between SOX9 and other molecular players, providing insights into the intricate signaling networks in fibrosis. Studies involving clinical patients, employing organoids and personalized medications, could customize treatments according to individual SOX9 profiles, thereby enhancing treatment outcomes and minimizing side effects, ultimately improving prognosis of patients.
